# Viral load at hospitalization is an independent predictor of severe COVID‐19

**DOI:** 10.1111/eci.13882

**Published:** 2022-10-12

**Authors:** Hjalmar Waller, Noelia Carmona‐Vicente, Axel James, Melissa Govender, Francis R. Hopkins, Marie Larsson, Marie Hagbom, Lennart Svensson, Helena Enocsson, Annette Gustafsson, Åsa Nilsdotter‐Augustinsson, Johanna Sjöwall, Johan Nordgren

**Affiliations:** ^1^ Division of Molecular Medicine and Virology, Department of Biomedical and Clinical Sciences Linköping University Linköping Sweden; ^2^ Division of Infectious Diseases, Department of Medicine Karolinska Institute Stockholm Sweden; ^3^ Division of Inflammation and Infection, Department of Biomedical and Clinical Sciences Linköping University Linköping Sweden; ^4^ Department of Infectious Diseases Vrinnevi Hospital Norrköping Sweden

**Keywords:** COVID‐19, hospitalization, independent predictor, SARS‐CoV‐2, severity, viral load

## 1 BACKGROUND

The coronavirus disease 2019 (COVID‐19) pandemic has put immense pressure on health care systems worldwide. Common symptoms of COVID‐19 include fever, myalgia, and cough, although the disease may develop into serious conditions causing acute respiratory distress syndrome (ARDS) or death.^1^, ^2^ Expanding our knowledge and use of prognostic factors for severe disease outcomes can facilitate clinical decisions for individualized treatment. Several risk factors have been described, with increased age and being overweight as two of the strongest predictors for severe COVID‐19 and death. In addition, male gender, cardiovascular diseases (CVD), and certain immunosuppressive conditions and therapies have been associated with increased risk for severe COVID‐19.^3^, ^4^, ^5^ Moreover, multiple inflammation markers in the blood have been shown to correlate to the outcome of severe acute respiratory syndrome coronavirus‐2 (SARS‐CoV‐2) infection, such as C‐reactive protein (CRP), which reaches high levels in patients with ARDS due to COVID‐19.^6^ Additional blood‐based biomarkers associated with disease severity include lymphocyte and neutrophil cell counts, interleukin (IL)‐1, IL‐6, IL‐10, and soluble urokinase plasminogen activator receptor (suPAR).^7^, ^8^, ^9^

ABO blood groups, as well as secretor status that determines the expression of blood group antigens in the mucosa, have been implicated as risk factors for susceptibility to SARS‐CoV‐2 infection. However, their effect on COVID‐19 disease severity remains unclear.^10^, ^11^

In 2003, during the SARS‐CoV epidemic, an association between a high viral load at hospital admission and worse prognosis was observed.^12^ Concerning SARS‐CoV‐2, there is relatively limited data on the correlation between viral load and disease severity, and studies have reported divergent results.^13^, ^14^ Several research groups have reported a higher viral load at time of hospitalization as well as longer duration of virus shedding^15^ in patients with severe COVID‐19.^13^, ^14^, ^15^, ^16^ However, other studies have not found such associations,^17^ or that while viral load was higher in severe cases, it was not a statistically significant predictor of disease severity.^18^

The aim of this study was to investigate the association between SARS‐CoV‐2 viral load, ABO blood groups and secretor status, and disease severity in a well‐defined cohort of hospitalized COVID‐19 patients^7^ and to further elucidate whether real‐time PCR cycle threshold values can be used as a predictive marker in clinical settings.

## 2 METHODS

This study was part of a prospective study of patients admitted to the Department of Infectious Diseases at Vrinnevi Hospital in Norrköping, Sweden between August 2020 and May 2021.^7^ In total, 62 patients were included in the cohort based on the following inclusion criteria: age ≥ 18 years, ability to provide consent, and a current COVID‐19 diagnosis verified through qPCR on throat or nasopharyngeal swab samples at time of admittance to hospital, using Abbott Real‐Time SARS‐CoV‐2 or Alinity m SARS‐CoV‐2 assays (Abbott, Solna, Sweden). Exclusion criteria were cognitive impairment and not speaking Swedish or English. Oral and written informed consent was obtained from all participants. The study protocol was approved by the Swedish Ethical Review Authority (Decision number 2020–02580). At inclusion, patients were asked to answer a questionnaire including symptom duration and smoking habits. Additional data were collected from digital medical records: body mass index (BMI), CVD, chronic pulmonary disease, chronic renal failure and diabetes, current medication, current immunosuppression (medical condition or therapy), length of hospital stay, highest level of care received, maximum need of oxygen supplementation, need of renal dialysis, and lastly, COVID‐19‐related medication (at the time; anticoagulants, remdesivir, and/or dexamethasone) (Table 1). The full cohort data pertaining to this study have been published previously.^7^ None of the patients had received any dose of COVID‐19 vaccination before study inclusion. Two patients declined to provide an additional nasopharyngeal swab sample for qPCR analysis at inclusion and were thus excluded from analysis.

**TABLE 1:** Clinical characteristics of the patient cohort included in this study. Adapted from Ref. 7

Patients, *n* = 60	
Male, *n* (%)	40 (67)
Age, median (range)	57.5 (32–91)
Body mass index (kg/m^2^), median (range)	30 (22–45)
Cardiovascular disease,^a^ *n* (%)	33 (55)
Chronic pulmonary disease, *n* (%)	15 (25)
Chronic renal failure, *n* (%)	7 (12)
Current or ex‐smoker, *n* (%)	33 (55)
Diabetes, *n* (%)	15 (25)
Immunocompromised^b^ at inclusion, *n* (%)	8 (13)
Symptom duration at inclusion, days median (range)	10 (2–30)
*COVID‐19 severity*	
Mild, *n* (%)	6 (10)
Moderate, *n* (%)	25 (42)
Severe, *n* (%)	21 (35)
Critical, *n* (%)	8 (13)

^a^ Includes hypertension.

^b^ Disease and/or on current immunosuppressant medicine, such as haematological or other malignancies, organ transplantation and rheumatic disease.

Patients were divided into four disease severity groups based on guidelines by the National Institutes of Health, USA, and approximated with respect to the highest level of care (pandemic department, intermediate or intensive care unit [ICU]) and the maximum need for oxygen therapy: mild (pandemic department, no oxygen supplement), moderate (pandemic department with oxygen supplement <5 L/min), severe (pandemic or intermediate care unit with oxygen supplement >5 L/min and ventilation assistance), and critical (ICU with or without mechanical ventilation).^7^ For statistical analysis, only two groups were compared: mild/moderate and severe/critical, due to the low number of patients in the mild and critical group, respectively.

Viral load was quantified from nasopharyngeal samples (*n* = 60) collected at study inclusion and kept at −80°C until analysis. RNA extraction was performed using the QIAamp Viral RNA Mini Kit (Qiagen, Hilden, Germany) according to the manufacturer's instructions. High‐Capacity cDNA Reverse Transcription Kit (Thermo Fisher Scientific, Uppsala, Sweden) was used for cDNA synthesis, following the manufacturer's instruction. Samples were run on a thermal cycler (S1000 Thermal Cycler, Bio‐Rad, Solna, Sweden) using the following protocol: 25°C for 10 min followed by 37°C for 2 h and finally 85° for 5 min. The qPCR was performed as previously described.^19^ In brief, samples were run in duplicates using iTaq Universal Probes Supermix (Bio‐Rad) with primers and probe targeting the SARS‐CoV‐2 envelope gene, as described by Corman et al.^20^ To allow for absolute quantification of viral load and inter‐assay validation, a standard curve with a serial dilution of a plasmid containing the genetic sequence for SARS‐CoV‐2 envelope protein was added to each qPCR run (pEX‐A128‐nCoV_E_Sarbecco, Eurofins Genomics, Solna, Sweden). Cycle threshold (*C*_t_)‐values (viral load) were divided into three categories: negative, *C*_t_ value >30 (low viral load), and *C*_t_ value <30 (high viral load). Cycle threshold values have an inverse relationship to viral load, that is, the lower the value in a sample, the higher the viral load. ABO blood group typing was performed at the laboratory of Clinical Immunology and Transfusion Medicine at Linköping University Hospital according to their standard operating procedures. Additionally, ABO blood group antigens and secretor phenotype in saliva were determined as previously described.^21^ Reporting of the study conforms to broad EQUATOR guidelines.^22^

## 3 STATISTICS

The distribution of all continuous variables in the study was tested by Shapiro–Wilk's test of normality. Variables to be included in the multivariate logistic regression were determined through univariate analysis either by Fisher's exact test for binary variables or Mann–Whitney *U*‐test for continuous variables. Any variable with a *p* < .1 in the univariate analysis was included in the final regression model. IBM SPSS Statistics version 27 was used for statistical analysis and calculations. For graphical illustrations, Graph Pad Prism 9.0.0 (GraphPad Software) was used.

## 4 RESULTS

**FIGURE 1:**
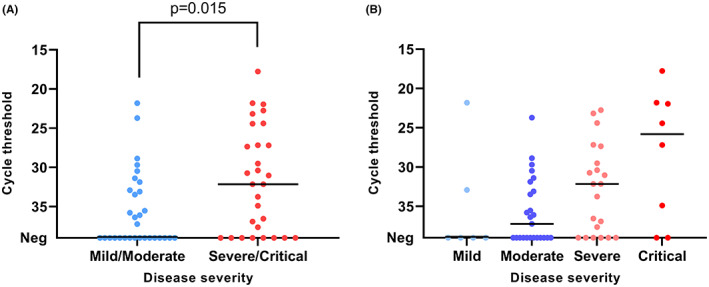
qPCR cycle threshold values for SARS‐CoV‐2 RNA in nasopharyngeal samples from patients at inclusion. The patients are stratified into different symptom severity groups (A: 2 groups; B: 4 groups). Comparison between the two groups was done using Mann–Whitney *U*‐test. The horizontal line represents the median, *n* = 60.

**FIGURE 2:**
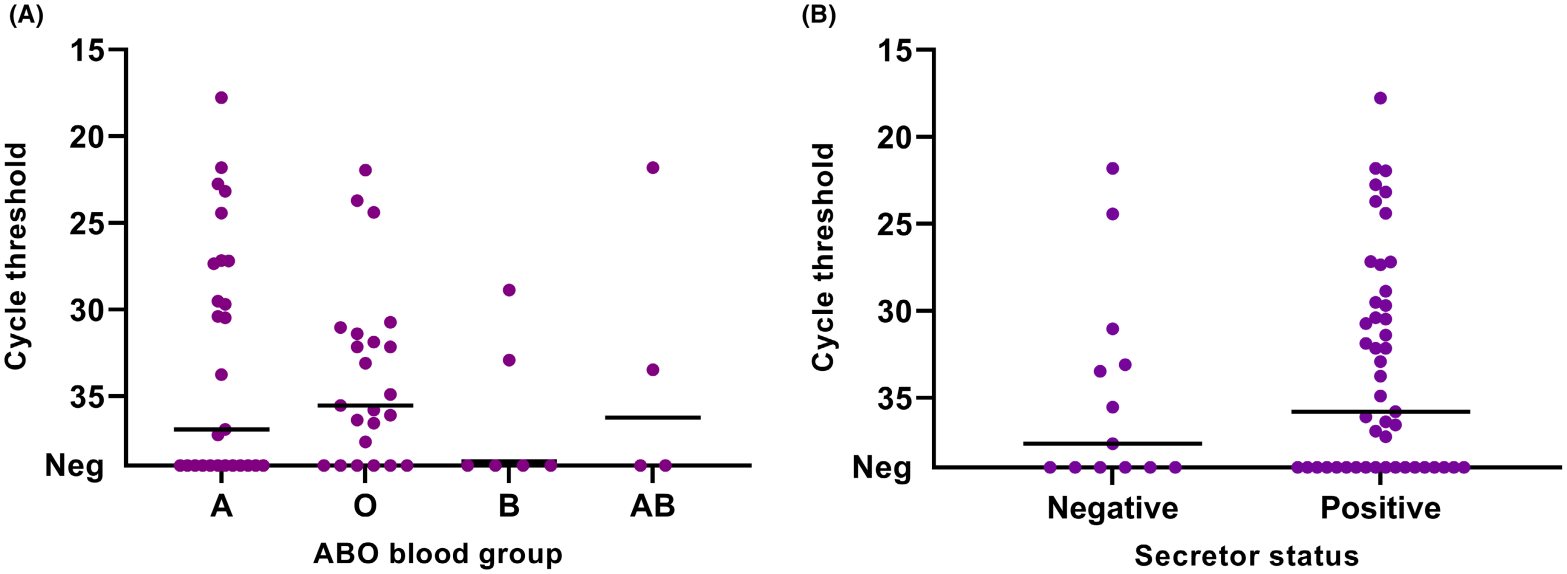
qPCR cycle threshold values for SARS‐CoV‐2 RNA in patient nasopharyngeal samples at inclusion. The patients are stratified by ABO blood groups (A) and secretor status (B). The horizontal line represents the median. *n* = 60.

At inclusion, SARS‐CoV‐2 viral load in the nasopharyngeal samples was higher (*p* = .015) in patients with more severe disease outcome (Figure 1A). The same association was observed in the four‐group analysis (mild, moderate, severe, critical), but the sample number was too low in the mild and critical groups to allow for a reliable statistical comparison (Figure 1B). There was no association between ABO blood groups or secretor status and disease severity (Table 2), or viral load at inclusion (Figure 2).

**TABLE 2:** Distribution of ABO blood groups and secretor status in hospitalized patients with COVID‐19, stratified by mild/moderate and severe/critical disease.

	Total number	Mild/moderate	Severe/critical
		*n* (%)	*n* (%)
ABO blood group			
A	29	14 (48)	15 (52)
0	23	11 (48)	12 (52)
B	6	4 (67)	2 (33)
AB	4	2 (50)	2 (50)
Secretor status			
Positive	48	23 (48)	25 (52)
Negative	14	8 (57)	6 (43)

To investigate whether viral load was an independent predictor of disease severity, a multivariate logistic regression was performed (Table 3). Potential co‐predictors were identified in univariate analyses. Neither age, gender, nor duration of symptoms before inclusion showed any association to severity or viral load at inclusion in the univariate analysis (*p* > .3). BMI, CRP, suPAR, and neutrophil‐to‐lymphocyte ratio (NLR) were identified as potential factors at the *p* < .1 criterium and were included in the logistic regression. Additionally, 35% (21/60) of the participants had received antiviral treatment (remdesivir) ≥ 1 day before study inclusion, which had a negative correlation with viral load (*p* = .1). As such, remdesivir treatment was also included as a potential confounder.

**TABLE 3:** Association between SARS‐CoV‐2 viral load as determined with qPCR cycle threshold (*C*_t_) values and COVID‐19 disease severity

Variable	Mild/moderate symptoms, *n* (%)	Severe/critical symptoms, *n* (%)	OR (95% CI)	aOR^a^ (95% CI)	*p*‐Value
Neg qPCR (Ref) *n* = 24	16 (52)	8 (28)	1.0 (Ref)	1.0 (Ref)	
*C*_t_ value >30 *n* = 21	11 (36)	10 (35)	1.82 (0.55–6.1)	4.78 (0.90–25.3)	.066
*C*_t_ value <30 *n* = 15	4 (13)	11 (38)	5.50 (1.32–22.9)	15.30 (1.74–134)	.014

^a^ Adjusted for remdesivir treatment, body mass index, soluble urokinase plasminogen activator receptor, C‐reactive protein, and neutrophil‐to‐lymphocyte ratio.

The multivariate regression analysis showed that high viral load (*C*_t_‐value <30, OR 15.3, *p* = .014) independently predicted COVID‐19 severity (Table 3).

## 5 DISCUSSION

In the present study, we observed that SARS‐CoV‐2 viral load at inclusion was higher in patients with more severe COVID‐19. In addition, viral load was shown to be an independent predictor of disease severity in this well‐characterized patient cohort.

Several previous studies have investigated the association between SARS‐CoV‐2 viral load and severity of COVID‐19, but the results are conflicting. Comparison between these studies is difficult as they vary to a large extent with regards to cohort composition, study design, sampling time‐points after onset of symptoms and sample selection.^13^ The results can also vary depending on host factors, for example one study reported a positive correlation of viral load with disease severity in younger age groups (<60 years), but not in older ones.^23^ However, a more consistent association between higher viral load and disease severity is found in cohorts of hospitalized patients^13^, ^15^, ^16^, ^24^ such as ours, although not always in multivariate analysis.^18^ This was also observed during the previous SARS‐CoV epidemic, where Chu et al. showed that high viral load at hospital admittance was independently associated with more severe forms of SARS.^12^ Thus, all these observations suggest that viral load at admittance to hospital could be a useful clinical predictor of COVID‐19 outcome. A previous study with influenza virus observed that individuals receiving higher doses of virus developed more severe symptoms.^25^ Higher viral load has also been associated with clinical symptoms and disease severity for several other viral infections, caused by, for example, metapneumovirus,^26^ respiratory syncytial virus^27^ and norovirus,^28^ but results are conflicting between studies.^29^, ^30^ One potential explanation for the observed associations between high viral load and disease severity is that higher virus replication and/or longer persistence and dissemination in the host, due to failure to control the viral infection, leads to more severe outcomes through dysregulated and/or overactive immune responses.^31^ Higher viral load might also indirectly reflect other comorbidities such as immunosuppression, which can affect the disease outcome.^32^ Nevertheless, the underlying mechanisms and pathophysiology connecting viral load and disease severity are not clear and require a deeper understanding.

We did not observe any effect of blood group antigens or secretor status on disease severity nor viral load at inclusion. Several studies have observed a lower infection susceptibility for individuals with blood group O. Still, any putative effect of blood group antigens on disease severity remains unclear.^10^ Few studies have investigated the role of secretor status, an important susceptibility factor for many viruses infecting the mucosa, in COVID‐19,^11^ and the results are conflicting.^33^, ^34^ The underlying mechanisms behind the observed findings of blood groups and risk of COVID‐19 are not fully understood, but several hypotheses have been proposed. These include a facilitation of binding of the SARS‐CoV‐2 virion to blood group A epitopes in the respiratory tract,^35^ or inhibition of the SARS‐CoV‐2 virion by natural anti‐A and anti‐B antibodies in individuals with blood group O.^36^ Support for the latter hypothesis comes from an observation that ABO antibody levels were lower in COVID‐19 patients than controls^37^ and from a study observing decreased risk of transmission between individuals with ABO incompatibility.^36^

Another hypothesis originates from the observation that individuals with blood group O have lower levels of von Willebrand factor,^38^ which thus could lower the risk of thrombosis during COVID‐19.^10^, ^39^

A strength of this prospective study is the well‐defined, although small, cohort of hospitalized patients with COVID‐19.^7^ The study cohort reflects some of the well‐known characteristics of severe disease, including co‐morbidities, predominance of male gender and obesity,^7^ although the only medical condition associated with disease severity in the multivariate analyses was being overweight. There was no effect of age, a well‐known risk factor. Reasons for this might include the limited number of patients in each group as well as the exclusion of patients with cognitive impairment, which is more common among the elderly. Another limitation of the study is the exclusion of patients not speaking Swedish or English which may potentially have skewed the cohort distribution. Moreover, some of the patients were initially receiving care at another pandemic department and were not included in the study until they were transferred to the Department of Infectious Diseases. This is reflected in the observation that 40% of the patients were negative for SARS‐CoV‐2 by qPCR in nasopharyngeal samples at study inclusion, despite having previously tested positive at hospital admission. However, the self‐reported symptom duration before study inclusion was not associated with disease outcome or viral load and as such was not a confounding factor. In addition, 35% of the patients had received antiviral treatment (remdesivir) before study inclusion and viral load quantification. However, this potential confounder was accounted for by inclusion in the multiple regression analysis. Performing the same analysis without adjusting for remdesivir treatment yielded similar results with high viral load (*C*_t_ < 30) independently predicting disease severity (OR 7.48, *p* = .038).

Additionally, it is important to point out that the results cannot be applied with certainty to the latest SARS‐CoV‐2 variants. The patients were included from the fall of 2020 to the spring of 2021, during which time the alpha variant was predominant in Sweden. A further, important limitation of using qPCR cycle threshold values is that they cannot be readily compared between studies or laboratories, as they depend on a multitude of factors, ranging from sample collection and nucleotide extraction to molecular tests used. Thus, the specific cut‐off for “high” and “low” viral load may influence the prognostic value of the variable and should be evaluated independently.

To conclude, we observed higher SARS‐CoV‐2 viral load in patients with a more severe disease outcome. Additionally, using multivariate analysis, we observed that the qPCR cycle threshold value was an independent predictor of disease severity in this cohort. As such, viral load may be a potential prognostic factor for disease outcome in patients hospitalized due to COVID‐19 and may contribute to effective targeted treatment of patients with higher risk of developing severe disease.

## FUNDING INFORMATION

This study was supported by Region Östergötland (ALF Grant, RÖ935411 (JS)); Regional ALF Grant 2021 (ÅN‐A and JS), Vrinnevi Hospital in Norrköping. Swedish Research Council project grant 201701091 (ML), SciLifeLab/KAW COVID‐19 Research Program (ML).

## CONFLICT OF INTEREST

The authors declare no conflict of interest.
